# Association between duration of neoadjuvant hormonal therapy and prostate-specific antigen response and pathological outcomes in high-risk prostate cancer undergoing robot-assisted radical prostatectomy

**DOI:** 10.3389/fonc.2026.1817402

**Published:** 2026-04-20

**Authors:** Jingsuo Wang, Yunfeng Wu, Chuanao Zhang, Jun Ouyang, Fan Zhang, Zhiyu Zhang

**Affiliations:** 1 The First Affiliated Hospital of Soochow University, Department of Urology, Suzhou, Jiangsu, China; 2 The First People’s Hospital of Jintan, Department of Urology, Changzhou, Jiangsu, China; 3 The First Affiliated Hospital of Soochow University, Department of Nephrology, Suzhou, Jiangsu, China

**Keywords:** extraprostatic extension, high-risk prostate cancer, neoadjuvant androgen deprivation therapy, neoadjuvant therapy duration, robot-assisted radical prostatectomy

## Abstract

**Purpose:**

The optimal duration of neoadjuvant hormonal therapy (NHT) for high-risk prostate cancer remains controversial. In this study, we evaluated the association between NHT duration and prostate-specific antigen (PSA) kinetics as well as pathological outcomes in patients who underwent extraperitoneal robot-assisted radical prostatectomy.

**Methods:**

We retrospectively analyzed the data of 72 patients with high-risk prostate cancer who received NHT followed by extraperitoneal robot-assisted radical prostatectomy with pelvic lymph node dissection at a tertiary hospital in 2025. Patients were stratified into the short-term (NHT, ≤3 months; n=41) and long-term (NHT, >3 months; n=31) groups. PSA response, pathological features, and perioperative outcomes were compared between the two groups.

**Results:**

The PSA nadir was significantly lower in patients who underwent long-term NHT than in those who underwent short-term NHT (median 0.02 vs. 0.23 ng/mL). The proportions of patients in whom PSA levels of <0.1 ng/mL (67.74% vs. 29.27%) and <0.2 ng/mL (83.87% vs. 41.46%) were achieved were significantly higher in the long-term group. Pathological analyses demonstrated a significantly lower incidence of extraprostatic extension in patients undergoing long-term NHT (38.71% vs. 70.73%). Operative time and intraoperative blood loss were similar between the groups.

**Conclusion:**

In patients with high-risk prostate cancer, undergoing NHT for longer than 3 months was associated with greater suppression of PSA and more favorable pathological outcomes. Particularly, we observed a reduced incidence of extraprostatic extension and no increase in perioperative morbidity. Achieving a substantial PSA response during NHT may be useful as an indicator for optimizing the timing of surgical intervention.

## Introduction

Prostate cancer is one of the most commonly diagnosed malignancies among men and, despite definitive local treatment, patients with high-risk disease face a substantial risk of biochemical recurrence, local progression, and distant metastasis (1–4). Radical prostatectomy represents a key treatment for localized prostate cancer; however, surgical monotherapy in patients with high-risk disease is frequently associated with adverse pathological features, particularly extraprostatic extension (EPE) and positive surgical margins (PSM), which are well-established predictors of oncological failure (5, 6).

To address these challenges, neoadjuvant hormonal therapy (NHT) has emerged as an adjunctive strategy to surgical intervention. The theoretical rationale for using NHT lies in its ability to suppress the androgen signaling axis, thereby reducing the tumor volume, lowering the clinical stage (downstaging), and potentially eradicating micrometastatic foci (7, 8). Early randomized controlled trials demonstrated that NHT could significantly improve pathological outcomes, particularly by reducing PSM rates (9, 10). However, in these early studies, pathological improvements did not consistently result in a survival benefit, leading to a period of skepticism regarding the routine use of NHT in clinical guidelines.

Recently, the neoadjuvant landscape has evolved with the introduction of next-generation androgen receptor signaling inhibitors (ARSIs), including apalutamide, darolutamide, and enzalutamide (6, 11–13). Contemporary evidence indicates that deeper suppression of prostate-specific antigen (PSA) and more pronounced pathological downstaging may be achieved using intensified and prolonged neoadjuvant androgen blockade than can be achieved using conventional androgen deprivation therapy alone (12, 14, 15). Although a 3-month neoadjuvant regimen is commonly adopted in traditional protocols, emerging data indicate that in a substantial proportion of patients with high-risk disease this duration may be insufficient for achieving maximal therapeutic response.

Despite these advances, substantial uncertainty remains regarding the optimal duration of NHT and its relationship with maximal pathological benefit. Existing studies are characterized by heterogeneous treatment protocols and limited representation of contemporary robotic-assisted surgical platforms; thus, the incremental value of prolonged NHT on adverse pathological features such as EPE and PSM remains incompletely defined (16). Moreover, although a decline in the PSA level is widely used as a surrogate marker of treatment response, the clinical relevance of achieving profound PSA suppression while neoadjuvant therapy is being administered—particularly in relation to pathological downstaging and surgical timing—has not been fully elucidated (14).

Therefore, in this study, we retrospectively evaluated the association between the duration of NHT (≤3 months vs. >3 months) and biochemical as well as pathological outcomes in a contemporary cohort of patients with high-risk prostate cancer who underwent extraperitoneal robot-assisted radical prostatectomy with pelvic lymph node dissection. By systematically analyzing PSA kinetics, pathological downstaging, and surgical margin status, we aimed to provide real-world evidence to inform individualized surgical timing and optimize multimodal treatment strategies for high-risk prostate cancer.

## Materials and methods

### Study design and ethical approval

This retrospective cohort study included consecutive patients with high-risk prostate cancer who underwent neoadjuvant hormonal therapy followed by extraperitoneal robot-assisted radical prostatectomy at the Department of Urology, the First Affiliated Hospital of Soochow University between January and December 2025. Clinical and pathological data from this period were retrospectively collected and analyzed to evaluate the impact of NHT duration. We stratified patients according to the duration of preoperative NHT into short-term (≤3 months) and long-term (>3 months) groups. The 3−month threshold was selected because a 3−month course of neoadjuvant androgen deprivation therapy has historically been the most commonly used duration in early randomized studies evaluating neoadjuvant therapy prior to radical prostatectomy, and it therefore serves as a practical reference point for comparing conventional short-course therapy with prolonged treatment (17).

The study protocol was reviewed and approved by the Institutional Review Board of the First Affiliated Hospital of Soochow University (protocol number 226/2026). The study was conducted in accordance with the Declaration of Helsinki and its later amendments. Given the retrospective nature of the study design, the requirement for written informed consent was waived. All patient data were anonymized prior to analysis.

### Patient selection

Patients were eligible for inclusion if they had (1) prostate adenocarcinoma that was histologically confirmed through transrectal or transperineal needle biopsy; (2) disease that was classified as high-risk prostate cancer according to the D’Amico criteria, namely, defined by the presence of at least one of the following features: clinical stage T2c or higher, biopsy Gleason score of ≥8, or initial PSA level of >20 ng/mL (18); (3) undergone NHT prior to surgery; and (4) subsequently undergone treatment with extraperitoneal robot-assisted radical prostatectomy and pelvic lymph node dissection at our institution.

The exclusion criteria were: (1) evidence of distant metastasis on preoperative imaging; (2) receipt of other neoadjuvant treatments such as chemotherapy or radiotherapy; (3) a history of other pelvic malignancies or major pelvic surgery; and (4) incomplete clinical or pathological data, particularly regarding PSA kinetics or the status of surgical margins.

### Neoadjuvant therapy and surgical technique

All patients received NHT prior to surgical intervention. The specific NHT regimens were categorized into two types: (1) combined androgen blockade, which consisted of androgen deprivation therapy (ADT) using luteinizing hormone-releasing hormone (LHRH) agonists (e.g., goserelin 10.8 mg or leuprorelin 11.75 mg every 12 weeks) combined with bicalutamide (50 mg once daily); and (2) ADT combined with ARSIs, which involved ADT plus a next-generation ARSI, such as apalutamide (240 mg once daily), darolutamide (600 mg twice daily), abiraterone acetate (1,000 mg once daily), or enzalutamide (160 mg once daily). The choice of regimen was determined by the treating physician based on contemporary clinical guidelines, individual disease characteristics, and patient preference. In patients with high-risk features, intensified androgen receptor pathway inhibition using next-generation androgen receptor signaling inhibitors (ARSIs) in combination with ADT may be considered, as emerging evidence suggests that this strategy may achieve deeper PSA suppression and improved pathological responses in the neoadjuvant setting (19, 20). Therefore, the selection of ADT alone with combined androgen blockade or ADT plus an ARSI reflected real-world clinical decision-making during the study period.

Surgical candidacy was reassessed after the neoadjuvant therapy was completed. Candidacy was based on comprehensive clinical evaluation and preoperative imaging findings, including multiparametric magnetic resonance imaging. Radical prostatectomy was considered when complete oncological resection was deemed technically feasible based on imaging findings; particularly, radical prostatectomy was considered as treatment for patients who initially presented with locally advanced disease (21, 22).

Following the designated period of NHT, all patients underwent robot-assisted radical prostatectomy using the Da Vinci Surgical System (Intuitive Surgical Operations, Inc., Sunnyvale, CA, USA). In our institution, robot-assisted radical prostatectomy is routinely performed using a standard extraperitoneal approach, and this approach was consistently applied to all patients included in the present study. Briefly, a preperitoneal space was created using a balloon dilator, and the robotic ports were placed without entering the peritoneal cavity. This approach was specifically chosen to minimize interference with the bowel and potential intraperitoneal complications. The surgical procedure included the radical resection of the prostate and seminal vesicles, followed by systematic dissection of the pelvic lymph nodes. All operations were performed by senior urologists with extensive experience in robotic surgery to ensure technical consistency and safety.

### Data collection and outcome measures

Clinical, laboratory, and pathological data were extracted from the electronic medical record system of our institution. The primary outcomes of interest were PSA kinetics and adverse pathological features following surgery. PSA response was evaluated using serial measurements during the period in which NHT was administered, with the PSA nadir defined as the lowest serum PSA level achieved prior to surgery. The proportions of patients in which PSA levels of <0.1 ng/mL and <0.2 ng/mL were achieved were also recorded.

Secondary outcomes included postoperative pathological characteristics and perioperative parameters. Radical prostatectomy specimens were independently reviewed by two experienced uropathologists according to standard criteria. Pathological assessments included pathological T stage, EPE, PSM, lymph node status, and pathological downstaging. The response to endocrine therapy was graded to assess the histological effect of treatment (23). Perioperative outcomes included operative time and estimated intraoperative blood loss.

### Statistical analysis

Statistical analyses were performed using R software (version 4.2.1; R Foundation for Statistical Computing, Vienna, Austria). Continuous variables were assessed for normality using the Shapiro–Wilk test. Normally distributed data are presented as mean ± standard deviation and were compared using the Student’s t-test. Non-normally distributed data are expressed as median and interquartile range and were compared using the Wilcoxon rank-sum test. Categorical variables are summarized as frequencies and percentages; they were compared using the chi-square or Fisher’s exact test, as appropriate. All statistical tests were two-sided, and a P value of <0.05 was considered statistically significant.

## Results

### Baseline characteristics

In total, 72 patients were included: 41 in the short-term group and 31 in the long-term group. The baseline clinical and demographic characteristics of the study population are summarized in **Table 1**. No statistically significant between-group differences were noted in terms of age, initial PSA level, clinical T stage, clinical N stage, the International Society of Urological Pathology (ISUP) grade, or neoadjuvant regimen type. The duration of NHT was significantly longer in the long-term group than in the short-term group, indicating adequate group separation for subsequent analyses.

**Table 1:** Baseline clinical and demographic characteristics of patients in the short-term and long-term groups who underwent neoadjuvant hormonal therapy.

Characteristic	Short-term group	Long-term group	P value
Number	41	31	
Age (years), mean ± SD	71.854 ± 5.5703	70.839 ± 5.2984	0.437
Initial tPSA (ng/mL), median (IQR)	32.45 (18.82, 104.24)	46.26 (25.87, 126.87)	0.197
Clinical T stage, n (%)			0.971
cT2c	12 (29.27)	8 (25.81)	
cT3a	4 (9.76)	4 (12.90)	
cT3b	16 (39.02)	12 (38.71)	
cT4	9 (21.95)	7 (22.58)	
Clinical N stage, n (%)			0.891
N0	31 (75.61)	23 (74.19)	
N1	10 (24.39)	8 (25.81)	
ISUP grade, n (%)			0.875
2	4 (9.76)	2 (6.45)	
3	8 (19.51)	8 (25.81)	
4	22 (53.66)	15 (48.39)	
5	7 (17.07)	6 (19.35)	
NHT regimen type, n (%)			0.761
CAB	20 (48.78)	14 (45.16)	
ADT+ARSI	21 (51.22)	17 (54.84)	
NHT duration (months), median (IQR)	3 (3, 3)	5 (4, 6)	<0.001

ADT, androgen deprivation therapy; ARSI, androgen receptor signaling inhibitor; CAB, combined androgen blockade; IQR, interquartile range; ISUP, International Society of Urological Pathology; NHT, neoadjuvant hormonal therapy; tPSA, total prostate-specific antigen; SD, standard deviation.

### PSA response

A significantly lower PSA nadir was achieved in patients who underwent long-term NHT than in those who underwent short-term NHT (**Table 2**). The proportions of patients in whom deep PSA suppression was achieved were significantly higher in the long-term group, including PSA levels of <0.1 ng/mL.

**Table 2:** Comparison of the prostate-specific antigen response between the short-term and long-term groups.

Characteristic	Short-term group	Long-term group	P value
Number	41	31	
PSA nadir after NHT (ng/mL), median (IQR)	0.23 (0.09, 0.57)	0.02 (0.01, 0.15)	<0.001
PSA response to <0.1 ng/mL, n (%)			0.001
No	29 (70.73)	10 (32.26)	
Yes	12 (29.27)	21 (67.74)	
PSA response to <0.2 ng/mL, n (%)			<0.001
No	24 (58.54)	5 (16.13)	
Yes	17 (41.46)	26 (83.87)	

IQR, interquartile range; NHT, neoadjuvant hormonal therapy; PSA, prostate-specific antigen.

### Pathological outcomes

The postoperative pathological outcomes are summarized in **Table 3**. The incidence of EPE was significantly lower in the long-term group than in the short-term group. No statistically significant between-group differences in pathological T stage distribution, T stage response, rate of lymph node metastasis, or rate of PSM were observed.

**Table 3:** Comparison of postoperative pathological characteristics between the short-term and long-term groups of patients who underwent neoadjuvant hormonal therapy.

Characteristic	Short-term group	Long-term group	P value
Number	41	31	
Postoperative pathological T stage, n (%)			0.804
pT0	2 (4.88)	3 (9.68)	
pT2	17 (41.46)	13 (41.94)	
pT3a	9 (21.95)	6 (19.35)	
pT3b	12 (29.27)	7 (22.58)	
pT4	1 (2.44)	2 (6.45)	
T stage response, n (%)			0.885
PD	4 (9.76)	3 (9.68)	
SD	16 (39.02)	11 (35.48)	
PR	19 (46.34)	14 (45.16)	
CR	2 (4.88)	3 (9.68)	
Extraprostatic extension, n (%)			0.007
No	12 (29.27)	19 (61.29)	
Yes	29 (70.73)	12 (38.71)	
Endocrine therapy response assessment grade, n (%)			0.517
0	2 (4.88)	2 (6.45)	
1	10 (24.39)	4 (12.90)	
2	19 (46.34)	12 (38.71)	
3	8 (19.51)	10 (32.26)	
4	2 (4.88)	3 (9.68)	
Lymph node pathology result, n (%)			0.492
Negative	32 (78.05)	22 (70.97)	
Positive	9 (21.95)	9 (29.03)	
Positive surgical margin, n (%)			0.611
No	31 (75.61)	25 (80.65)	
Yes	10 (24.39)	6 (19.35)	

CR, complete response; PD, progressive disease; PR, partial response; SD, stable disease.

### Correlation between EPE status and clinical outcomes

To further explore the clinical relevance of EPE following neoadjuvant therapy, patients were stratified according to postoperative EPE status (**Table 4**). Compared to patients with EPE, significantly higher rates of PSA suppression to <0.1 ng/mL and <0.2 ng/mL were achieved in those without EPE. Pathologically, patients without EPE were significantly more likely to have organ-confined disease (pT2) and to experience pathological downstaging. The incidence of PSM was markedly lower in patients without EPE than in those with EPE, and the grades of response to endocrine therapy were significantly more favorable.

**Table 4:** Comparison of prostate-specific antigen response and pathological characteristics between patients with and without extraprostatic extension.

Characteristic	With EPE	Without EPE	P value
Number	41	31	
PSA response to <0.1 ng/mL, n (%)			<0.001
No	30 (73.17)	9 (29.03)	
Yes	11 (26.83)	22 (70.97)	
PSA response to <0.2 ng/mL, n (%)			0.002
No	23 (56.10)	6 (19.35)	
Yes	18 (43.90)	25 (80.65)	
Postoperative pathological T stage, n (%)			<0.001
pT0	0 (0.00)	5 (16.13)	
pT2	7 (17.07)	23 (74.19)	
pT3a	14 (34.15)	1 (3.23)	
pT3b	17 (41.46)	2 (6.45)	
pT4	3 (7.32)	0 (0.00)	
T stage response, n (%)			0.027
PD	6 (14.63)	1 (3.23)	
SD	15 (36.59)	12 (38.71)	
PR	20 (48.78)	13 (41.94)	
CR	0 (0.00)	5 (16.13)	
Endocrine therapy response assessment grade, n (%)			0.011
0	3 (7.32)	1 (3.23)	
1	12 (29.27)	2 (6.45)	
2	18 (43.90)	13 (41.94)	
3	8 (19.51)	10 (32.26)	
4	0 (0.00)	5 (16.13)	
Positive surgical margin, n (%)			0.005
No	27 (65.85)	29 (93.55)	
Yes	14 (34.15)	2 (6.45)	

CR, complete response; EPE, extraprostatic extension; PD, progressive disease; PR, partial response; PSA, prostate-specific antigen; PSM, positive surgical margin; SD, stable disease.

### Perioperative outcomes

No significant differences between the short-term and long-term groups were observed in operative time or estimated intraoperative blood loss (**Table 5**).

**Table 5:** Comparison of perioperative outcomes between the short-term and long-term groups of patients who underwent neoadjuvant hormonal therapy.

Characteristic	Short-term group	Long-term group	P value
Number	41	31	
Operative time (minutes), mean ± SD	150.88 ± 35.137	158.84 ± 30.755	0.319
Intraoperative blood loss (mL), median (IQR)	55 (50, 70)	50 (45, 75)	0.900

IQR, interquartile range; SD, standard deviation.

### Subgroup analysis by NHT regimen

In both the combined androgen blockade and ADT plus ARSI subgroups, long-term NHT was consistently associated with a lower PSA nadir and higher rates of deep PSA suppression (**Table 6** and **7**). Notably, in the ADT plus ARSI subgroup, prolonged NHT was also associated with a significantly low incidence of EPE. No significant between-group differences in perioperative outcomes were observed.

**Table 6:** Comparison of oncological outcomes between short-term and long-term neoadjuvant hormonal therapy in patients receiving the combined androgen blockade regimen.

Characteristic	Short-term group	Long-term group	P value
Number	20	14	
PSA nadir after NHT (ng/mL), median (IQR)	0.295 (0.125, 0.8275)	0.06 (0.02, 0.1625)	0.018
PSA response to <0.1 ng/mL, n (%)			0.036
No	16 (80.00)	6 (42.86)	
Yes	4 (20.00)	8 (57.14)	
PSA response to <0.2 ng/mL, n (%)			0.013
No	14 (70.00)	3 (21.43)	
Yes	6 (30.00)	11 (78.57)	
Postoperative pathological T stage, n (%)			0.942
pT2	10 (50.00)	6 (42.86)	
pT3a	5 (25.00)	4 (28.57)	
pT3b	4 (20.00)	4 (28.57)	
pT4	1 (5.00)	0 (0.00)	
Extraprostatic extension, n (%)			0.163
No	5 (25.00)	7 (50.00)	
Yes	15 (75.00)	7 (50.00)	
Endocrine therapy response assessment grade, n (%)			0.338
0	2 (10.00)	1 (7.14)	
1	6 (30.00)	3 (21.43)	
2	10 (50.00)	5 (35.71)	
3	2 (10.00)	5 (35.71)	
Positive surgical margin, n (%)			1.000
No	16 (80.00)	12 (85.71)	
Yes	4 (20.00)	2 (14.29)	
Operative time (minutes), median (IQR)	145.5 (117.5, 158.75)	140.5 (125.75, 150)	0.944
Intraoperative blood loss (mL), median (IQR)	50 (38.75, 62)	64 (47, 80)	0.159

IQR, interquartile range; NHT, neoadjuvant hormonal therapy; PSA, prostate-specific antigen.

**Table 7:** Comparison of oncological outcomes between short-term and long-term neoadjuvant hormonal therapy in patients receiving the androgen deprivation therapy + androgen receptor signaling inhibitor regimen.

Characteristic	Short-term group	Long-term group	P value
Number	21	17	
PSA nadir after NHT (ng/mL), median (IQR)	0.17 (0.05, 0.38)	0.02 (0.01, 0.07)	0.019
PSA response to <0.1 ng/mL, n (%)			0.025
No	13 (61.90)	4 (23.53)	
Yes	8 (38.10)	13 (76.47)	
PSA response to <0.2 ng/mL, n (%)			0.034
No	10 (47.62)	2 (11.76)	
Yes	11 (52.38)	15 (88.24)	
Postoperative pathological T stage, n (%)			0.367
pT0	2 (9.52)	3 (17.65)	
pT2	7 (33.33)	7 (41.18)	
pT3a	4 (19.05)	2 (11.76)	
pT3b	8 (38.10)	3 (17.65)	
pT4	0 (0.00)	2 (11.76)	
Extraprostatic extension, n (%)			0.049
No	7 (33.33)	12 (70.59)	
Yes	14 (66.67)	5 (29.41)	
Endocrine therapy response assessment grade, n (%)			0.670
0	0 (0.00)	1 (5.88)	
1	4 (19.05)	1 (5.88)	
2	9 (42.86)	7 (41.18)	
3	6 (28.57)	5 (29.41)	
4	2 (9.52)	3 (17.65)	
Positive surgical margin, n (%)			1.000
No	15 (71.43)	13 (76.47)	
Yes	6 (28.57)	4 (23.53)	
Operative time (minutes), mean ± SD	156.71 ± 34.211	169.53 ± 30.541	0.237
Intraoperative blood loss (mL), mean ± SD	63.286 ± 18.69	56.353 ± 22.685	0.308

IQR, interquartile range; NHT, neoadjuvant hormonal therapy; PSA, prostate-specific antigen; SD, standard deviation.

## Discussion

In this retrospective cohort study, we investigated the association between the duration of NHT and biochemical as well as pathological outcomes in patients with high-risk prostate cancer undergoing extraperitoneal robot-assisted radical prostatectomy. Our findings demonstrate that extending NHT beyond 3 months is associated with a deeper PSA response and a significantly lower incidence of EPE. Importantly, these benefits were achieved without an increase in perioperative morbidity and were consistently observed across different neoadjuvant hormonal regimens.

Recent evidence has supported the potential role of neoadjuvant hormonal therapy before robotic prostatectomy. In a multicenter retrospective study, Dong et al. (24) evaluated patients with oligometastatic prostate cancer who received neoadjuvant hormonal therapy followed by robot-assisted radical prostatectomy and reported improved perioperative parameters and more favorable pathological outcomes compared with surgery alone. Although the study population consisted of patients with oligometastatic disease rather than high-risk localized disease, these findings support the concept that effective preoperative androgen suppression may facilitate tumor regression and improve surgical outcomes. Our findings further complement this evidence by suggesting that, even among patients with high-risk localized prostate cancer, the duration of neoadjuvant hormonal therapy may influence the degree of PSA suppression and the likelihood of achieving more favorable pathological features.

The pronounced effect of prolonged NHT on PSA kinetics observed in our study is biologically plausible and consistent with the concept of sustained suppression of the androgen receptor pathway (25). Prolonged androgen deprivation induces cumulative apoptotic effects and progressive metabolic suppression in androgen-dependent prostate cancer cells, although extended exposure may be required to achieve meaningful arrest of growth in more resistant tumor clones (26–30). This biological mechanism may partly explain the deeper PSA suppression observed in patients receiving longer NHT in our cohort.

The most clinically relevant pathological finding of this study was the significant reduction in EPE associated with prolonged NHT. Although no direct difference in PSM rates was observed between the short-term and long-term groups, our subsequent analyses demonstrated that an EPE-negative status was strongly associated with a markedly lower incidence of PSM. This suggests that the pathological benefit of prolonged neoadjuvant therapy may be primarily mediated through improved local confinement of the tumor rather than through a direct effect on surgical margin status. From a mechanistic perspective, extended androgen deprivation may promote regression of the tumor’s invasive front and reduce microscopic capsular penetration, thereby increasing the likelihood of organ-confined disease at the time of surgery (31–33). In this context, the lower PSM rate observed in patients without EPE likely reflects improved delineation of the surgical plane rather than a direct pharmacological effect of neoadjuvant therapy on margin biology.

Beyond the neoadjuvant setting, radical prostatectomy is increasingly being considered as part of a multimodal treatment strategy for patients with advanced-risk prostate cancer. In a recent study, Beyatlı et al. (34) compared perioperative complications and oncological outcomes between patients with high-risk and oligometastatic prostate cancer undergoing open radical prostatectomy and found no significant differences in complication rates or biochemical recurrence during mid-term follow-up. These findings suggest that surgical intervention may be feasible and clinically meaningful even in selected patients with metastatic or highly aggressive disease when performed in experienced centers. In this broader therapeutic context, optimizing preoperative systemic therapy—such as the duration and intensity of neoadjuvant hormonal treatment—may further enhance the effectiveness of surgery by improving local tumor control and pathological outcomes.

An exploratory finding of this study was the close association between profound PSA suppression during neoadjuvant therapy and favorable pathological features, particularly the absence of EPE. This observation supports the hypothesis that PSA kinetics during NHT may reflect the extent of intraprostatic tumor regression. Rather than adhering to a fixed duration of NHT, individualized timing of surgery that is guided by PSA response may represent a reasonable strategy in select patients with high-risk disease (14, 35). Given the retrospective nature of our analysis, PSA thresholds (e.g., <0.01 ng/mL) should be interpreted as hypothesis-generating rather than definitive criteria for clinical decision-making.

Our subgroup analyses further indicated that the biochemical advantages of prolonged neoadjuvant therapy were consistent across different hormonal regimens. Deeper PSA suppression was achieved with both combined androgen blockade and ADT plus ARSI regimens with longer treatment duration; however, the reduction in EPE observed in the ADT plus ARSI subgroup was more pronounced. This finding is consistent with emerging evidence that suggests that intensified inhibition of the androgen receptor pathway may enhance pathological response when inhibition is maintained for an adequate duration (11–13, 36).

Despite the encouraging findings, several limitations of this study must be acknowledged. First, this was a retrospective single-center study, which inherently introduces the potential for selection bias and unmeasured confounding despite the generally balanced baseline characteristics between the two groups. Second, the sample size of 72 patients, although sufficient for detecting significant differences in primary outcomes, may have limited the power of the subgroup analyses. Third, the present study focused primarily on short-term pathological and perioperative outcomes, and the duration of follow-up was insufficient to evaluate long-term oncological endpoints such as biochemical recurrence–free survival, metastasis-free survival, or overall survival. This limitation is particularly important in patients with high-risk prostate cancer, in whom long-term oncological control represents a key determinant of treatment success. Therefore, whether the pathological improvements observed with prolonged neoadjuvant therapy ultimately translate into meaningful survival benefits remains to be determined. Prospective studies with larger cohorts and extended follow-up are required to address this question. Fourth, a significant reduction in EPE was observed; however, the study was not powered to detect small differences in PSM rates between treatment durations. Finally, the study was conducted at a single high-volume center with surgeries performed by surgeons experienced in robotics; this may limit generalizing the PSM results to lower-volume settings.

## Conclusion

In patients with high-risk prostate cancer who underwent robot-assisted radical prostatectomy, NHT of 3 months or more was associated with deeper PSA suppression and more favorable pathological outcomes, particularly with regard to a lower incidence of EPE. These benefits were achieved without an increase in perioperative morbidity and were consistent across different neoadjuvant hormonal regimens. Although prolonged therapy was not directly associated with a reduced PSM rate, the strong relationship between EPE and margin positivity highlights the potential indirect pathological advantages of extended neoadjuvant treatment. Profound PSA suppression during neoadjuvant therapy may serve as a hypothesis-generating marker for individualizing surgical timing. Prospective, multicenter studies with long-term oncological follow-up are warranted to validate these findings and define the optimal duration of NHT in high-risk prostate cancer.

## Data Availability

The datasets presented in this study can be found in online repositories. The names of the repository/repositories and accession number(s) can be found below: https://doi.org/10.6084/m9.figshare.31343569.v1.
